# Human Cytomegalovirus Infection and Neurocognitive and Neuropsychiatric Health

**DOI:** 10.3390/pathogens13050417

**Published:** 2024-05-16

**Authors:** Shawn D. Gale, Thomas J. Farrer, Reagan Erbstoesser, Scott MacLean, Dawson W. Hedges

**Affiliations:** 1 The Department of Psychology, Brigham Young University, Provo, UT 84602, USA; mscottp@student.byu.edu (S.M.); dawson_hedges@byu.edu (D.W.H.); 2 The Neuroscience Center, Brigham Young University, Provo, UT 84602, USA; 3 Idaho WWAMI Medical Education Program, University of Idaho, Moscow, ID 83844, USA; tfarrer@uidaho.edu; 4 The Department of Biology, Brigham Young University, Provo, UT 84602, USA; rlerb615@student.byu.edu

**Keywords:** human cytomegalovirus (HCMV), congenital CMV, neurocognitive, neuropsychiatric, memory, dementia

## Abstract

A common infection, human cytomegalovirus (HCMV) has been associated with a variety of human diseases, including cardiovascular disease and possibly certain cancers. HCMV has also been associated with cognitive, psychiatric, and neurological conditions. Children with congenital or early-life HCMV are at risk for microcephaly, cerebral palsy, and sensorineural hearing loss, although in many cases sensorineural loss may resolve. In addition, HCMV can be associated with neurodevelopmental impairment, which may improve with time. In young, middle-aged, and older adults, HCMV has been adversely associated with cognitive function in some but not in all studies. Research has linked HCMV to Alzheimer’s and vascular dementia, but again not all findings consistently support these associations. In addition, HCMV has been associated with depressive disorder, bipolar disorder, anxiety, and autism-spectrum disorder, although the available findings are likewise inconsistent. Given associations between HCMV and a variety of neurocognitive and neuropsychiatric disorders, additional research investigating reasons for the considerable inconsistencies in the currently available findings is needed. Additional meta-analyses and more longitudinal studies are needed as well. Research into the effects of antiviral medication on cognitive and neurological outcomes and continued efforts in vaccine development have potential to lower the neurocognitive, neuropsychiatric, and neurological burden of HCMV infection.

## 1. Introduction

A beta herpesvirus [1], human cytomegalovirus (HCMV) is a common and widely distributed infectious disease. The estimated overall average worldwide prevalence of HCMV is approximately 83 percent, although there is significant regional variability [2], with Asia, Africa, and South America in general having higher HCMV seroprevalences than western Europe and North America [3]. However, most people infected with HCMV do not experience distinct symptoms, may be asymptomatic [4], or may present with non-specific symptoms seen in other viral illnesses such as those associated with mononucleosis including fever, headache, and fatigue [5]. HCMV infection may occur at any age and can be present at birth [1]. Further, HCMV has been associated with a range of diseases including cardiovascular disease and possibly cancer [5] and may aggravate or even increase risk for autoimmunity [6]. HCMV is particularly problematic in those who are immunocompromised or who have congenital infection [5]. Despite the high seroprevalence of HCMV, many women of childbearing potential are seronegative for HCMV and as such are at risk for contracting HCMV during pregnancy [3]. In short, the overall burden of HCMV on humanity has been described as “substantial” in terms of health and quality of life [7].

Numerous risk factors increase vulnerability to HCMV infection, including family poverty for children [8], increasing age [3], crowding, and an increased number of sexual partners [6]. Despite these risk factors, the degree of country economic development does not always correlate with the prevalence of HCMV seropositivity [3]. Following acute HCMV infection, the host will continue to harbor the virus in a latent state indefinitely [9]. Reactivation of the virus may occur for a variety of reasons including in response to psychological stressors [10]. The reactivation of HCMV in immunocompetent individuals typically results in no or mild symptoms, though it may be significant in some cases, and reactivation can be severe in the immunocompromised [9].

In addition to its relationships with cardiovascular disease and possibly with cancer [5], HCMV may be neurotropic in that it appears to infect and alter function and structure in differentiated neurons derived from human-induced pluripotent stem cells, although whether HCMV is neurotropic remains controversial [11]. Regardless of whether HCMV is neurotropic, however, it has been associated with a variety of neurological manifestations, including fetal brain injury [12], poor cognitive function in older adults [13], an increased risk for development of neurodegenerative diseases such as Alzheimer’s disease [14], and various neuropsychiatric disorders [15]. This review focuses on congenital, perinatal, and early-life HCMV infection and child development, HCMV and cognitive function in adults and older adults, associations between HCMV and dementia, and associations between HCMV and psychiatric disorders.

## 2. Congenital, Perinatal, and Early-Life HCMV Infection and Child Development

Worldwide, HCMV is the most common congenital infection [16]. Diagnosis can be difficult in that most babies with congenital HCMV are asymptomatic [17,18]. While a HCMV diagnosis can be made by analyzing blood, urine, or saliva postnatally [17], it is not routinely carried out prenatally except in circumstances that raise suspicion for congenital HCMV infection such as an abnormal ultrasound or flu-like symptoms in the mother or upon request from the mother [18]. According to the results of a meta-analysis using data from countries with universal screening for congenital HCMV, congenital HCMV infection is found in approximately 0.67 percent of all births globally. In low-income countries, the prevalence of congenital HCMV is higher, at 1.42 percent [19], which is concerning because perinatal and congenital HCMV infection can adversely affect a variety of aspects of subsequent child neurodevelopment. For example, in a large population-based sample from the United States, congenital HCMV increased the risk of microcephaly in newborns sevenfold [20]. In this same study, congenital HCMV was also associated with chorioretinitis and other ophthalmic abnormalities (1.9 percent versus 0.04 percent), hearing loss (2.1 percent versus 0.2 percent), and neonatal seizures (6.5 percent versus 0.2 percent) [20].

Congenital HCMV is thought to injure the central nervous system (that is, affect neurodevelopment) in many ways, such as through changes to progenitor cells, adversely affecting neuronal migration, and disrupting the blood brain barrier, as well as causing encephalitis or meningitis [21]. Through histological examination of fetal brain tissue, Piccirilli et al. demonstrated that HCMV may result in microglial activation, astrocytosis, vascular changes (e.g., endothelial hypertrophy), polymicrogyria, lissencephaly, and even necrosis to varying degrees [12]. The mechanism specifically related to sensorineural hearing loss is unclear but may be related to damage of stem cells or damage to inner ear structures such as the organ of Corti [22].

A meta-analysis investigating the association between congenital HCMV and cerebral palsy showed that the prevalence of cerebral palsy in patients with congenital HCMV was 26 percent. Conversely, among patients with cerebral palsy, 10.9 percent had congenital HCMV. Moreover, congenital HCMV was associated with a worse form of cerebral palsy than that found in cerebral palsy patients without congenital HCMV [23,24]. Similarly, there is evidence that preterm children with early postnatal HCMV infection may differ from preterm children without infection on neuroimaging measures of structural and functional brain connectivity [25,26] and that preterm children who became infected with HCMV in the early postnatal period demonstrate cognitive impairment when evaluated as adolescents [27].

In their meta-analysis of children with otherwise asymptomatic congenital HCMV, Bartlett et al. [28] found that 7 to 11 percent had sensorineural hearing loss, although in up to half of these cases resolution of the sensorineural hearing loss occurred. Bartlett et al. [28] further found that, on average, the included studies suggested no difference between children with and without hearing loss on available neurodevelopmental measures. Still, research is ongoing, and the long-term effects of congenital HCMV are unclear. For example, in a group of children approximately 7.5 years old with a history of asymptomatic congenital cytomegalovirus infection, 17 percent of whom had some hearing loss and were being followed to determine long-term hearing outcomes, approximately 30 to 45 percent demonstrated impairment compared to typically developing children on measures of vestibular function, gaze, and balance [29]. Investigating neurodevelopmental outcomes, with the last evaluation occurring most commonly between ages 3 and 12 months but in some cases at age 72 months, a prospective cohort study in Belgium of 753 children with congenital HCMV infection found normal neurodevelopmental outcomes in 70.4 percent of the children but mild, moderate, or severe impairment in the remaining 29.6 percent. Notably, impairment occurred in both symptomatic and asymptomatic groups. The authors further reported that 46 percent of the children had hypotonia, although many of these underwent improvement, 10.4 percent had mild to severe cognitive impairment, and 17.2 percent had sensorineural hearing loss [16].

A longitudinal prospective study investigated associations between postnatal early-life infection with HCMV and subsequent cognitive outcomes [30] found in adjusted models associations between total intelligence quotients and early-childhood HCMV at age 8 years (but not at age 15 years), with attentional control at age 8 years, and with reading comprehension at age 9 years, although none of the other investigated cognitive outcomes were associated with early-life HCMV. Although these findings suggest that early-life HCMV infection may be associated with some cognitive impairment, they also suggest that these associations might not persist beyond childhood. Based on findings from their scoping review of 33 studies investigating associations between congenital HCMV and neurodevelopmental outcomes, Pesch et al. [31] concluded that congenital HCMV is commonly associated with adverse neurodevelopmental outcomes while noting the limited generalizability of the studies due to inconsistent definitions of asymptomatic and symptomatic congenital HCMV, a lack of studies controlling for socioeconomic standing and maternal education, and hard classification boundaries.

Additional research is needed to better understand the effects of congenital and early-childhood HCMV on brain development. Areas requiring further study include better estimates of the number of children seropositive for HCMV who have deficits in brain function and the identification of factors associated with outcomes in children with congenital HCMV or early-life infection with HCMV. In addition, some studies indicate that improvement in neurological and neurocognitive outcomes can occur in children with congenital or early-childhood HCMV [16,28,30], underscoring the importance of identifying not only factors associated with HCMV but also with neurological and neurocognitive improvement in children infected with HCMV.

## 3. HCMV and Cognitive Functioning in Adults and Older Adults

HCMV infection has been associated with deficits in cognitive function in adults and in older adults in both cross-sectional and longitudinal studies, although not all findings have been consistent. Understanding the association between HCMV and cognitive function in adults is important, particularly as it relates to characterizing whether HCMV is a risk factor for dementia.

In one longitudinal study investigating the association between HCMV and cognitive function, Aiello et al. [32] found that higher levels of HCMV antibodies were associated with an increased rate of cognitive decline. In a study of 849 older adults, HCMV seropositivity was associated both with Alzheimer’s disease and amplified cognitive decline over five years [14]. In contrast, in another study of older adults, there were no baseline differences by seropositivity or differences in cognitive decline over 18 months between groups seropositive and seronegative for HCMV [33]. In a large cohort study (N = 5617) of adults over 65 years of age from the Health and Retirement Study, Stebbins et al. [34] found that HCMV seropositivity and serointensity were numerically associated with decreased cognitive function but that this association was not statistically significant in the fully adjusted models. Associations between HCMV and cognitive function have also been evaluated in older adults with other diseases. Findings from these studies suggest that HCMV seropositivity might interact with certain other diseases to adversely affect cognitive function. In a study of older adults from the Health and Retirement Study who had survived cancer, HCMV seropositivity was not associated with cognitive function, but the HCMV seropositive group that also had high levels of inflammation had worse cognitive function than the group that was both HCMV seronegative and had low levels of inflammation [35], suggesting that interactions between HCMV and immune function might affect cognitive function. In a study based on data from adults aged 85 years or older from the Tokyo Oldest Old Study, the association between HCMV and decreased cognitive function was only found in the group with carotid atherosclerosis [36]. Similarly, HCMV was associated with worsened cognitive function in schizophrenia [37]. Overall, these findings suggest that other medical conditions such as atherosclerosis and advanced age might affect associations between HCMV and cognitive function, although these relationships may be complex in that, for example, it has been suggested that HCMV infection may contribute to the development of atherosclerosis [5], increased risk for cardiovascular disease [38], and even intracranial arterial stenosis [39]. Finally, it is important to consider potential interactions between infections and overall health and how these interactions may manifest in aging, with its associated changes in physiological function [40], and the subsequent correlations with functional and cognitive outcomes [41].

Overall, findings from studies investigating associations between HCMV and cognitive function in older adults have been inconsistent, with some studies showing an association and others not showing associations between cognitive function and HCMV in older adults. Some findings suggest that HCMV might interact with other diseases to adversely affect cognitive function.

Less work has been done to identify potential associations between HCMV and cognitive functioning in middle-aged or younger adults than has been done in older adults. In a study that included adults as well as children aged 6 to 16 years (average age 11.1 years), HCMV was not associated with math or reading scores, attention span, or visuospatial function in adjusted models [42]. Similarly, in those participants aged 60 years and older, there were no associations between HCMV seropositivity and performance on measures of memory or executive function. In contrast, Tarter et al. [42] did find that HCMV seropositivity was associated—albeit with small effect sizes—with worse performance on a measure of processing speed (odds ratio 1.41) and with more errors on a learning test (odds ratio 1.43) in adults aged 20 to 59 years. In a cross-sectional study, Dickerson et al. [43] examined cognitive function in 521 adults with an average age 32.8 years and found that HCMV antibody serointensity was correlated with worse performance on a measure of delayed recall, working memory, and visuospatial function but not learning, set-shifting, attention, language function, or visuomotor speed. In contrast to these findings, a study in adults between 40 and 70 years of age did not find an association between HCMV seropositivity or antibody concentrations and cognition, and there was not an interaction with age [44].

In general, findings regarding associations between HCMV and cognition in adults appear to be more consistent in older adults than they are in middle-aged adults. In addition, there appears to be less research in young and middle-aged adult populations compared to older ones, but most research on HCMV and cognition has been carried out in children [7]. It is significant that an increasing number of studies appear to be considering the impact of multiple infections on cognition [45]. Given that HCMV has been associated with increased risk for medical conditions that may independently affect cognition, including heart disease and stroke [38], it seems necessary that future studies control for and consider these conditions. Similarly, as noted below, HCMV is also associated with mental health conditions such as depression, which also, by itself, could contribute to cognitive function. However, most studies consider either associations between HCMV and mood or HCMV and cognition, but not both. Furthermore, most studies investigating associations between cognitive function in adults are cross-sectional, and additional longitudinal studies are sorely needed to confirm these associations as well as to elucidate mechanisms of action [45]. In addition, identification of risk factors for the occurrence of deficits in cognitive function in adults and older adults with HCMV infection is needed.

## 4. HCMV and Dementia

Numerous large-scale population studies have demonstrated an association between various infectious agents and an increased risk of dementia and cognitive decline [13,46]. Although there are discrepancies in the research literature, HCMV specifically may also increase the risk of dementia. Understanding the possible role HCMV in dementia is particularly salient given that the population prevalence of HCMV increases with age and that immunosenescence increases the prevalence of opportunistic infections among older adults and reinfection related to HCMV [47].

Some studies have examined the underlying pathology of HCMV as a mechanism for the potential role of HCMV in dementia. In a murine model, one study demonstrated that the presence of CMV increased the phosphorylation of tau and resulted in a greater degree of tau pathology [48]. This is supported by a human post-mortem study that utilized serum, cerebrospinal fluid, and other tissues to explore the presence of biomarkers of AD [49]. In this study, HCMV antibodies were associated with neurofibrillary tangles, with increased beta-amyloid and with the proinflammatory cytokine interferon-γ, which is known to play a role in AD.

Although these are important findings, the clinical implications are unclear when considering a cross-sectional study that demonstrated that those with Alzheimer’s disease versus controls were no more likely to have HCMV (36 percent of sample compared to 35 percent) [50]. Conversely, a cross-sectional study demonstrated that HCMV seropositivity was higher among those with AD (89 percent) relative to healthy controls (75 percent), with an odds ratio of 2.33 [51]. As noted, Barnes and colleagues [14] examined the baseline seropositivity of HCMV among older adults (average age of 76 years), with a follow-up period of 5 years. At follow-up, analysis revealed that HCMV seropositivity resulted in an increased incidence of Alzheimer’s disease with a relative risk ratio of 2.15. In other words, those with the virus were more than twice as likely to develop Alzheimer’s disease than those who were HCMV seronegative. Additionally, those with HCMV demonstrated a more rapid rate of cognitive decline than those who were seronegative for HCMV. In another study, the baseline seropositivity of HCMV in 850 older adults was used to examine longitudinal changes in multiple cognitive domains [13]. This study demonstrated that in the HCMV seropositive group, greater declines were observed in memory and visuospatial function relative to those who were seronegative. Stebbins et al. [34] also demonstrated that HCMV and a higher IgG antibody response were associated with reduced cognitive function, but only among individuals with lower educational attainment.

Using electronic medical records, Lee and colleagues [52] demonstrated that the presence of HCMV was associated with a greater risk of dementia (odds ratio of 1.9), with a possible differential impact on vascular dementia (odds ratio of 2.9) relative to Alzheimer’s disease (odds ratio of 1.6). These findings are consistent with those of Kawasaki et al. [36], who demonstrated an interaction between carotid artery stenosis and HCMV in which those with both HCMV IgG titers and greater stenosis had lower cognitive function on a mental status screener. Additionally, a post-mortem study explored the relative proportions of various viral pathogens in the brain samples of individuals previously diagnosed with vascular dementia, finding that HCMV was present in 14 of 15 individuals with vascular dementia (93 percent) but in only 10 of 29 (34 percent) of individuals without vascular dementia [53].

In contrast to the above findings, multiple studies have failed to find an association between HCMV and dementia risk. In one cross-sectional study (n = 102), there was no difference in HCMV antibody levels between a group of older adults with AD versus the healthy control [54]. Lopatko Lindman et al. [55] also failed to identify a correlation between serum beta-amyloid and HCMV antibodies in a group of older adults with AD. Additionally, Zilli et al. [56] used the Framingham Heart Study cohorts to explore the risk of dementia in the presence of multiple infectious agents, including HCMV. Their analysis revealed no association between chronic infection and incident dementia. In a twenty-year longitudinal study, George et al. [57] tracked changes to neuropsychological function based on seropositivity of multiple infections, including HCMV, with no apparent risk identified in those with the virus. Similarly, Torniainen-Holm et al. [58] tracked cognitive function in HCMV seropositive individuals stratified by age, and while there was an initial indication of lower test performance for those with HCMV, these effects disappeared after correcting for multiple tests. Lastly, Lövheim et al. [59] suggest that HCMV by itself may not increase the risk of Alzheimer’s disease but rather that there may be an interaction between HCMV and herpes simplex virus 1 (HSV-1), in that the presence of both resulted in a significant increase in the risk of AD (odds ratio of 5.66). Similarly, Vivek et al. [35] also demonstrated that HCMV alone is not associated with cognitive impairment in a cohort of cancer survivors but that the interaction of HCMV and higher levels of inflammatory biomarkers seems to result in lower cognitive function than inflammation alone.

These discrepancies in findings from studies investigating associations between HCMV and dementia have been addressed in a few systematic reviews. A 2019 meta-analysis explored several human herpes viruses and the risk of mild cognitive impairment and dementia, but also found mixed results. The authors suggested that the source studies were of low quality. Additionally, they suggested that most source studies do not show strong support for an association between HCMV and dementia, although serum IgG CMV seropositivity and IgG titers were associated with dementia and cognitive decline in a few source studies [60].

Two more recent studies have also attempted to address these discrepancies in the research literature. A comprehensive review by Sanami et al. [61] suggested that while HCMV appears to promote immune and inflammatory processes that are associated with biomarkers of Alzheimer’s disease, HCMV is more likely a risk factor for Alzheimer’s disease and not a causal factor per se. Furthermore, Ji et al. [62] used meta-analytic methods to examine the potential association between HCMV and Alzheimer’s disease. The overall analysis showed only a weak and non-statistically significant relationship, with an odds ratio of 1.33 (confidence interval of 0.88 to 2.03), although this overall finding included substantial heterogeneity. Upon further post-hoc analyses of subgroups, Ji et al. [62] found that HCMV had a strong association with AD in East Asian samples (OR = 2.39), cohort studies (OR = 1.99), and studies in which confounding variables were controlled (2.05).

As is the case with childhood and adult HCMV infection, cognitive function outcomes among studies related to HCMV and risk for dementia are mixed. It is unclear whether HCMV by itself or perhaps in combination with other infections and/or medical conditions increases dementia risk or if HCMV is simply present but is not necessarily a contributing factor to the neurodegenerative process. It has been suggested that HCMV is not causative of dementia in the overall population but may contribute to cognitive decline due to individual vulnerability (e.g., genetics, disease, immune-related issues, etc.) [34,58]. Careful study of factors associated with risk for both HCMV infection and risk of dementia, such as lower socioeconomic status [4,63], must be pursued. As such, identification of risk factors for dementia in the context of HCMV infection is needed, as are longitudinal studies investigating long-term outcomes of HCMV. An additional question concerns the effects of antiviral medication on long-term cognitive outcomes in HCMV infection.

## 5. Psychiatric Disorders Associated with HCMV

In addition to its possible associations with sensorineural hearing loss, cognitive deficits, and dementia, accumulating evidence suggests that HCMV may be associated with a variety of psychiatric disorders, including mood disorders, anxiety disorders, autism-spectrum disorders, and schizophrenia, further illustrating the range of effects that HCMV might have on the brain. As an example of HCMV being associated with psychiatric disorders, results from the Danish Blood Donor Study found an association between HCMV seropositivity and the category of all the International Classification of Diseases 10 diagnostic codes for psychiatric disorders [15]. 

### 5.1. Mood and Related Disorders

In a study based on the Danish Blood Donor Study, Burgdorf et al. [15] additionally found possible associations between HCMV seropositivity and mood disorders, their category of “neurotic, stress-related, and somatoform disorders”, and their category of “suicide or suicide attempt” (p. 154) [15].

In patients with major depressive disorder, HCMV seropositivity was associated with lower gray-matter volume, including volume reduction in orbitofrontal, temporal, and parietal regions, and HCMV seropositivity was related to decreased resting-state connectivity in sensorimotor and salience networks [64]. Furthermore, those with major depressive disorder who are also seropositive for HCMV appear to have decreased white-matter integrity in tracts connecting the salience and executive networks compared to those with major depressive disorder who are seronegative for HCMV [65]. In a similar fashion, studies in bipolar disorder have suggested associations between infection with HCMV and reduced brain volumes, including in mesial temporal lobe volume and its structures such as the hippocampus and dentate gyrus [66]. However, Zheng and Savitz [66] point out the variability in HCMV associations with both major depressive disorder and bipolar disorder and brain volumes, though perhaps with more evidence in major depressive disorder, as well as that most studies in healthy participants seropositive for HCMV do not find associations with brain volume. However, associations between behavior or mood and HCMV infection have been found. For example, in persons seropositive for HCMV, depression was reported in those with higher antibody levels in both cross-sectional [67,68] and longitudinal studies [69,70]. Other studies have linked higher HCMV antibodies and suicide attempts in those with major depressive disorder [71,72,73]. Interestingly, since a few studies did not find that these associations were related to some inflammatory markers (e.g., interleukin-6 and C-reactive protein), it is possible that HCMV infection itself, rather than overall inflammation, underlies the association between depression [69,70,74] or suicidality [71] and higher HCMV levels. However, Zheng et al. [75] found that HCMV seropositivity was associated with bipolar disorder (odds ratio: 2.45) and major depressive disorder (odds ratio: 3.70) and suggested that these findings were consistent with the hypothesis that reactivation of HCMV leads to the neuroinflammation associated with some psychiatric disorders.

### 5.2. Anxiety Disorders

In their community-based sample of older adults, Phillips et al. [68] found HCMV serointensity was associated with anxiety in the HCMV seropositive group. However, in a longitudinal Finnish sample of 6,250 adults 30 to 65 years of age at baseline who were evaluated 11 years later, seropositivity for HCMV did not confer an increased risk for new-onset anxiety. In fact, HCMV at baseline appeared to be associated with a reduced risk for the presence of generalized anxiety disorder but not for other anxiety disorders at follow-up [76].

### 5.3. Autism-Spectrum Disorders

A prospective study by Keymeulen et al. [16] in Belgium found that 2.6 percent of their study participants with HCMV had an autism-spectrum disorder compared to a prevalence of autism-spectrum disorder in Flanders of 0.6 to 0.7 percent. The young age of the children—62.5 percent were between ages 4 and 12 months at the time of their last evaluation—precludes knowing whether the autism-spectrum findings persist into later childhood and adolescence. In contrast, a two-sample Mendelian randomization study found no relationship between HCMV and autism-spectrum disorder [77].

### 5.4. Schizophrenia

Perhaps one of the most studied mental illnesses associated with HCMV is schizophrenia, a psychotic disorder that often includes disordered thought, behavior, and emotions, as well as impairment in neurocognitive function. HCMV has been associated with the risk for development of this disorder, with levels of functioning both behaviorally and cognitively, and with measures of brain structure [32,66,78,79]. Nevertheless, the heterogeneity in this research literature is significant. For example, one study found that higher levels of HCMV may worsen the impact of schizophrenia on cognitive functioning [37], consistent with findings from Houenou et al. [79] showing that in people with schizophrenia, HCMV was related to decreased performance on the California Verbal Learning Test, a measure of verbal episodic memory. Furthermore, Houenou et al. [79] also found that HCMV was associated with decreased volume of the right hippocampus in patients with schizophrenia, consistent with a prior study that found associations between clinical outcomes and right hippocampal volume [80]. Evidence for etiological implications of HCMV for schizophrenia includes the observation that HCMV and schizophrenia often afflict similar low-socioeconomic populations, early signs of schizophrenia may be associated with higher levels of HCMV antibodies, and treatments for HCMV may treat schizophrenia to some extent [78]. Given this evidence for a significant association, the absence of typical HCMV-related encephalitis in schizophrenia patients suggests that HCMV may not causally contribute to the development of schizophrenia [78].

As is the case with findings investigating associations between HCMV and developmental, cognitive, and dementia outcomes, results from studies looking at relationships between HCMV and psychiatric disorders are often inconsistent. Clearly, additional work is needed to better characterize the associations between HCMV infection and mood disorders and anxiety disorders, including identification of risk factors (e.g., socioeconomic status, stressful life events, genetic predisposition) associated with the development of psychiatric disorders in the context of HCMV infection.

## 6. Perspectives and Conclusions

A detailed description of potential mechanisms underlying the effects of HCMV infection on the central nervous system is beyond the scope of this review. As outlined by Zheng et al. [64], however, HCMV could affect the brain via several non-mutually exclusive mechanisms: direct cellular damage due to reactivation of latent HCMV virus infection in the brain, inflammatory processes due to reactivation of latent HCMV, tissue damage from HCMV-induced autoimmunity, and HCMV-induced immune modulation. Furthermore, immediate early protein 2, characteristic of CMV infection, could contribute to cognitive dysfunction by a mechanism of diminished long-term potentiation, reduced synaptic plasticity protein expression, and reduced dendritic spine density [81]. We have mentioned other potential mechanisms in the sections above including those associated with changes to the cerebrovascular system. Furthermore, latent HCMV infection can reactivate, and it is quite likely that HCMV infection may interact in unknown and possibly detrimental ways with other newly acquired or latent viral, bacterial, and even other pathogens [41,59,82].

The findings reviewed here indicate that HCMV can be associated with neurological, psychiatric, and cognitive abnormalities across the lifespan, although findings are often inconsistent, with many studies not findings associations between HCMV and neurological, cognitive, and psychiatric outcomes. In view of the potential severity of these possible HCMV-associated deficits, it is important to address the reported inconsistencies in outcome studies to identify risk and protective factors for and against the development of brain dysfunction in people seropositive for HCMV. Further, careful attention to interactions between HCMV and infection by other infectious diseases, geographical regions, potentially confounding variables, and other methodological considerations including the use of large longitudinal samples are needed to better delineate and clarify relationships between HCMV and cognitive, psychiatric, and neurological outcomes including dementia. Identification of and attention to protective and risk factors against and for adverse effects from HCMV could decrease the overall neurological, psychiatric, and cognitive burden of HCMV.

Given that HCMV may be associated with a range of neurological, psychiatric, and cognitive deficits and infects a large proportion of the worldwide population, vaccine development will be important to decrease HCMV infection. Similarly, even if a vaccine does not prevent infection, it may mitigate negative outcomes. Despite ongoing vaccine development for HCMV, however, no licensed vaccine for HCMV is currently available [1], although development and distribution of a vaccine for HCMV would result in significant progress in public health. In fact, the US National Institute of Medicine considers the development of a vaccine for HCMV of critical importance [1].

In addition to vaccine development, screening newborns for HCMV could result in earlier detection of perinatal and congenital HCMV [19], possibly enabling implementation of interventions such as antiviral treatment and speech and language treatment to decrease the adverse effects of perinatal and congenital HCMV [17]. Universal screening for congenital HCMV can be expensive, and debate regarding its utility [17] is beyond the scope of this review. However, recent studies have suggested that the necessity for screening varies by location, with low- and middle-income countries having an estimated congenital HCMV prevalence approximately three-times higher than that in high-income countries [19]. Furthermore, the efficiency and sensitivity of screening methods has been an issue, although recent studies, such as one using a pooled saliva test method, have demonstrated encouraging results [83].

In conclusion, HCMV is associated with a range of adverse cognitive, psychiatric, and neurological outcomes and substantially contributes to the burden of brain disease, although currently available findings are often inconsistent. The discrepant research findings underscore the need for consistently applied definitions of symptomatic and asymptomatic congenital HCMV [31], careful use of covariates including socioeconomic status, maternal and educational attainment [31], previous infections, geographical regions, host immune factors, study methodology, and HCMV strain to better characterize associations between HCMV and human brain function. Research investigating the effects of antiviral treatment on HCMV outcomes [31], ongoing vaccine development, and application of public-health measures have the potential to reduce the potentially large burden of brain dysfunction due to HCMV.
